# Inappropriate data selection and statistical method lead to overestimated mortality for hospitalised HIV/AIDS patients

**DOI:** 10.1017/S0950268820001363

**Published:** 2020-06-22

**Authors:** J. Wang

**Affiliations:** Julius Center for Health Sciences and Primary Care, University Medical Center Utrecht, Utrecht, the Netherlands

Dear Editor,

Recently, a simple-to-use nomogram for predicting the survival of hospitalised HIV/AIDS patients was published by Yuan *et al*. as ‘Development and external-validation of a nomogram for predicting the survival of hospitalised HIV/AIDS patients based on a large study cohort in western China’ [1]. The authors claimed the nomogram had high performance in external validation and was clinically useful. However, two serious issues observed in the model development and validation process may lead to unreliable predictions and could cause more harm than benefit.

In the paper, it said ‘The survival of the training cohort was 94.8% and 90.8% at the 10-day and 20-day, respectively’ [1], indicating the 10-day mortality is around 5% and 20-day mortality is about 9%, which are incredibly high. Compared to the numbers in the article they cited [2], the overall in-hospital mortality rate was only 29.36 per 100 person-years [2]. Such high mortality rate cannot be explained by their discussion and was actually caused by the inappropriate method used in data selection and statistical analysis in this study.

In the data selection process, the authors selected the latest admission for those patients with multiple admissions, thus excluded 2892 admissions (around one-third of the total admissions) [1]. However, it is obvious that death can only happen in the last admission and all the excluded previous admissions had no event. By excluding these event-free observations, the in-hospital mortality was artificially upward biased.

Moreover, in the statistical analysis, the authors used the standard Cox model without considering discharge as a competing risk. Discharged patients were no longer at risk of in-hospital death, thus simply censoring them at time of discharge will overestimate the in-hospital mortality [3]. Actually, this model provided a prediction in a virtual world where patients can either die in the hospital or stay in the hospital forever, which is not useful for clinical practice in real-world [4].

Both issues mentioned above lead to overestimation of in-hospital mortality and the predicted 10-day and 20-day survival probabilities were not correct. These cannot be found in the temporal validation performed by the authors since they made the same mistakes there as well. So the model performance in external validation was not reliable.

The nomogram proposed in this study should be carefully checked by the authors and further assessed by independent researchers before using by clinicians for decision making.
